# The direct anterior approach in total hip arthroplasty: Publication trends of Asian countries on PubMed

**DOI:** 10.1016/j.amsu.2020.06.001

**Published:** 2020-06-04

**Authors:** Sholahuddin Rhatomy, Faiz Alam Rasyid, Krisna Yuarno Phatama

**Affiliations:** a Department of Orthopaedics and Traumatology, Dr Soeradji Tirtonegoro General Hospital, Klaten, Indonesia; b Faculty of Medicine, Public Health and Nursing, Universitas Gadjah Mada, Yogyakarta, Indonesia; c Soeradji Tirtonegoro Sport Center and Research Unit, Dr Soeradji Tirtonegoro General Hospital, Klaten, Indonesia; d Department of Orthopaedics and Traumatology, Dr Saiful Anwar General Hospital, Malang, Indonesia; e Faculty of Medicine, Brawijaya University, Malang, Indonesia

**Keywords:** Direct anterior approach (DAA), Total hip replacement (THR), PubMed, Hip, Database search, Asia

## Abstract

**Background:**

Total hip replacement (THR) is one of the most successful surgical treatment for advanced hip osteoarthritis. Some surgical approaches for THR have been established, one of it is Direct Anterior Approach (DAA), which is a relatively new and less commonly used, especially in Asian countries. This review aims to consolidate information from PubMed on the direct anterior approach (DAA) for total hip replacement (THR) in Asian countries.

**Purpose:**

To collect and consolidate information from PubMed on the total hip replacement (THR) using the direct anterior approach (DAA) in Asian countries.

**Methods:**

A search on the PubMed was done for DAA for THR. There were 461 search results about the DAA for THR publications obtained in total, and 51 articles meet the criteria were analyzed for details. The details include top countries publishing the topics, number of publications per year, top 5 journal publishing the DAA for THR topics, top 5 first authors publishing the articles on this topic, and top 5 author on all position published this topic.

**Results:**

The Journal of Arthroplasty was the leading publisher on this topic, with 10 articles published. Author Yasuhiro Homma, Tomonori Baba, and Kazuhiro Oinuma published the most number with 9 articles as one of the authors. Japan is the leading country for the publication on this topic with 34 published journals in total.

**Conclusion:**

The number of published articles in Asian country per year is still inconsistent, with one year without any publication on this topic. Searching the data on the DAA for THR in PubMed bestow useful information about good sources of publication on this topic.

## Highlights

• Direct anterior approach.
• Total hip arthroplasty.
• Publication trends.
• Asian countries.

## 1 Introduction

Total hip replacement (THR) is one of the most successful surgical treatment for advance hip osteoarthritis [1,2]. This method is responsible for relieving pain, improving gait, restoring function, and increasing quality of life in patient with degenerative joint disease of the hip [[1], [2], [3]]. Kurtz et al. [4] reported a 50% increase in the prevalence of THR from 1990 to 2002.

The common surgical approach techniques currently used for THR are lateral approach, postero-lateral approach, and antero-lateral approach. Lateral approach is the most popular one [3,5]. The other surgical approach for THR includes posterior, lateral, antero-lateral, and direct anterior approach (DAA) [6]. Almost all of THR approaches must be done with capsule and muscle splitting and dissection to open the surgical field well. This procedure may lead to severe post-operative pain and joint instability [7].

The direct anterior approach (DAA) is a relatively new technique for total hip replacement and less commonly used, especially in Asian Countries [1]. Considering that DAA is an inter-muscular and inter-nervous technique, this approach has less soft tissue damage [8,9]. Many reports have shown the benefits of this approach compared to others, which are less post-operative pain, lower dislocation rate, and earlier mobilization, thus becoming more popular among surgeons in Asian countries [2,3,9]. However, DAA may requires longer learning curve and special instruments [2].

Based on the experience of some surgeons, that the anthropology of Asian populations does not seem exactly the same as European, triggering the emergence of a more frequent research on DAA in Asian countries in some recent years. However, there are no studies that have conducted systematic reviews on the Asian's DAA research trends. This study is aimed to provide the overview on the DAA for THR publications in Asian countries from PubMed that were analyzed to find the details about the literature.

PubMed is a database developed by National Center for Biotechnology Information (NCBI) as one of the division of the U.S. National Library of Medicine (NLM) under National Institute of Health (NIH). MEDLINE is the main structure of the PubMed, as primary database of the NLM that focused on Biomedicine topic. PubMed was launched in 1996 and undergo significant design update in 2000 and 2002 [10].

PubMed provide more than 28 million publications in biomedicine. On average, there are about 2.5 million PubMed daily users, they run about 3 million searches and 9 million page views on a daily average [11].

## 2 Methods

The following strategy was used: the terms used to search on the search engine were “direct anterior approach total hip replacement”. Preferred Reporting Items for Systematic Reviews and Meta-Analyses (PRISMA) guidelines were used to perform the comprehensive data collection [12]. A bibliometric evaluation was done to all the search results.

This research includes all type of studies: surgical technique, clinical study, cadaveric study, review article, systematic review, and meta-analysis. The studies above were searched regardless of the language used in the publications. Microsoft Excel was used to create a database, the demographic data from PubMed were loaded, analyzed, and visualized using this software.

## 3 Results

**Fig. 1:**
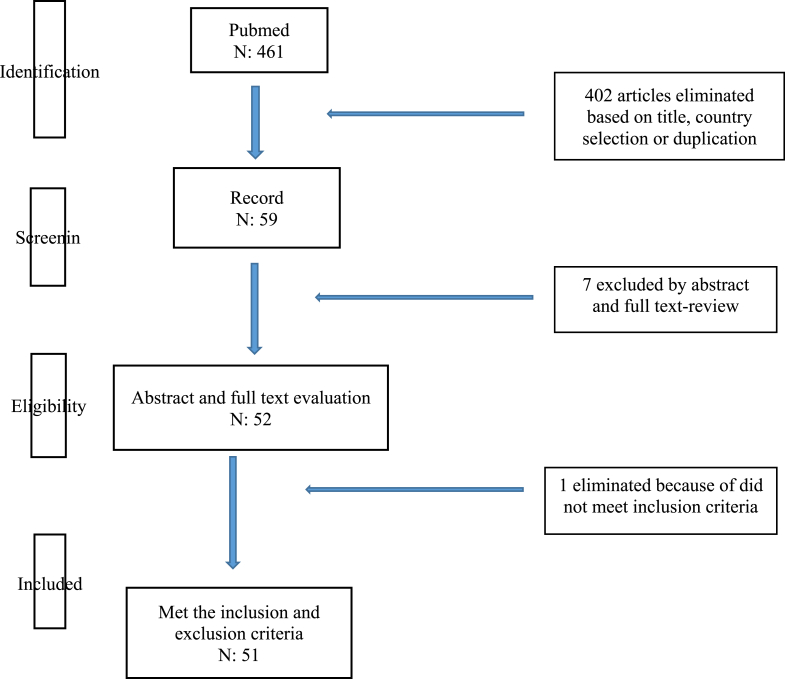
PRISMA Flow diagram of the article selection process.

There were 461 search results obtained in total. From that number, only 59 articles were published by Asian countries (12.8%) and they were going further evaluation. Seven search results were not related to the issues of direct anterior approach for total hip replacement. Furthermore, articles categorized as letter to editor were found in 1 search result. The other 51 remaining articles related to direct anterior approach for total hip replacement published by Asian center were eligible to be included for further evaluations after title, abstract, and full-text screening (Fig. 1).

**Fig. 2:**
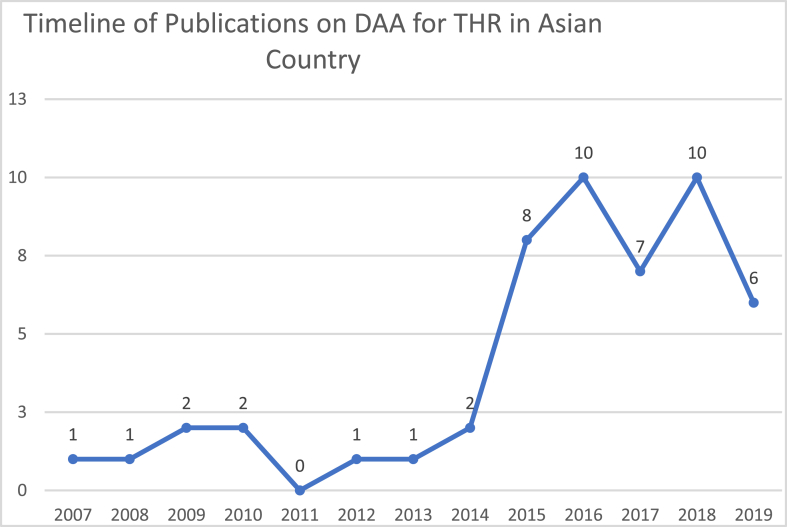
Number of DAA for THR publication in Asian countries by year.

Fig. 2 shows the yearly distribution of publication. The number of published articles per year was 3.92 averagely. If the study period is divided into the publication before 2014 and 2014 and after, the yearly average number of publication was 1.1 before 2014, and 7.1 from 2014 onward. There is an upward trend of publication from 2011 to 2016, but the number fell in 2017. There are two peaks of publication with 10 published article per year, that was in 2016 and 2018. However, the number was in insignificant trend on the number of publication per year after 2016, but from 2012 onward, there are no years without publication.

### 3.1 Journals

**Fig. 3:**
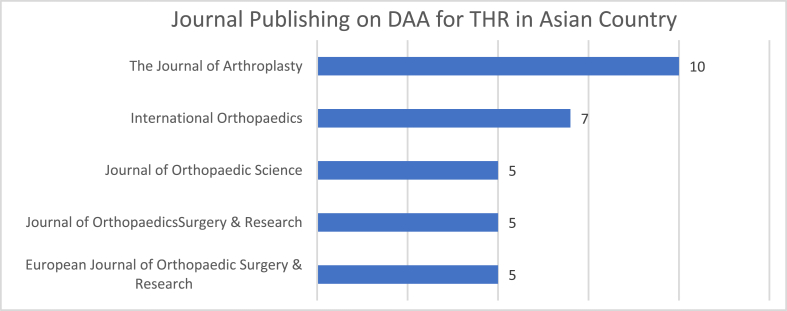
Top 5 journals publishing DAA for THR publication in Asian countries.

Fig. 3 shows the top 5 journals publishing on the DAA for THR in Asian countries. The Journal of Arthroplasty was the leading publisher on this topic, with 10 articles published. The Journal of Arthroplasty published 19% of all articles on the DAA for THR topics in Asian countries. Number, quality, and impact factor of the journal publishing on the DAA for THR in Asian countries are shown in Table 1. There are 18 journal publishers included in this study, 6 of them have impact factor <1, and 12 of them have impact factor >1. There are 8 journals which are Q1 journal, 7 journals are Q2, and the rest is Q3 or lower.

**Table 1:** List of the most popular journals publishing DAA for THR in Asian countries.

No	Journal	Number of Article	Impact Factor	Quality
1	Arch Orthopaedic Trauma Surgery	2	1.17	Q1
2	BMC Musculoskeletal Disorders	3	1.52	Q2
3	Chinese Orthopaedic Association	1	1.33	Q1
4	Clinical Orthopaedic and Related Research	2	4.09	Q1
5	European Journal of Orthopaedic Surgery & Traumatology	5	0.76	Q2
6	HIP International	3	0.51	Q2
7	International Orthopaedics	7	1.5	Q1
8	Journal of Orthopaedic Science	5	1.25	Q2
9	Journal of Orthopaedic Surgery and Research	5	1.90	Q2
10	Medical Principles and Practice	1	1.50	Q2
11	Medicine	1	0.92	Q2
12	Operative Orthopaedic and Traumatology	1	0.28	Q4
13	Orthopaedic Clinic of North America	1	1.84	Q-
14	Orthopaedic Research and Reviews	1	0.75	Q4
15	Orthopaedics & Traumatology: Surgery & Research	1	0.83	Q1
16	PLOS One	1	1.95	Q1
17	The Journal Of Arthroplasty	10	1.73	Q1
18	The Journal of Bone and Joint Surgery	1	1.97	Q1

### 3.2 Country

**Fig. 4:**
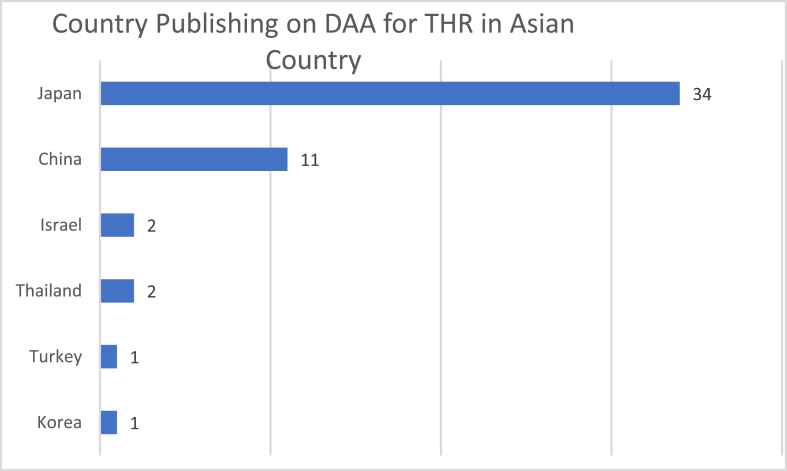
Countries with DAA for THR publication in Asia.

Fig. 4 shows the country publishing the articles about DAA for THR in Asian countries. Japan is the leading country for the publication on this topic. Japan published 34 journals in total, from 2007 to 2019. China is the second leading country with 11 journals published. Japan and China centers published 88% from the total number of publication on DAA for THR topics in Asian countries.

### 3.3 Author

There were a total of 211 unique authors, of whom 8 authors wrote more than 5 publications. Among the first authors as listed on studies, only two authors wrote five or more publications as first author. Author Yasuhiro Homma, Tomonori Baba, and Kazuhiro Oinuma published the most number with 9 articles as one of the authors.

**Fig. 5:**
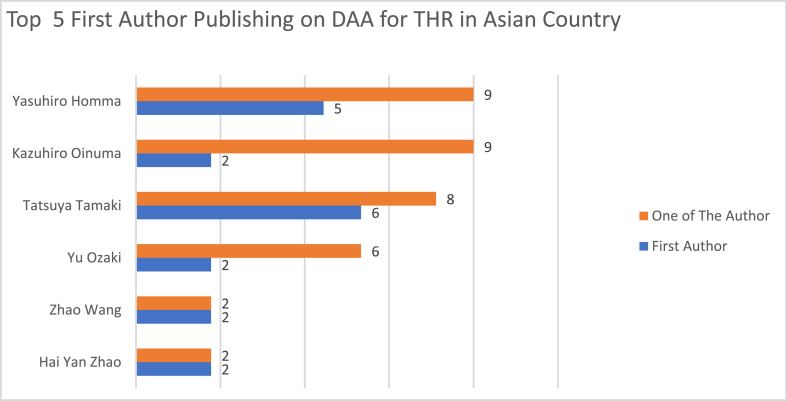
Top 5 first authors with most DAA for THR publication in Asian countries.

**Fig. 6:**
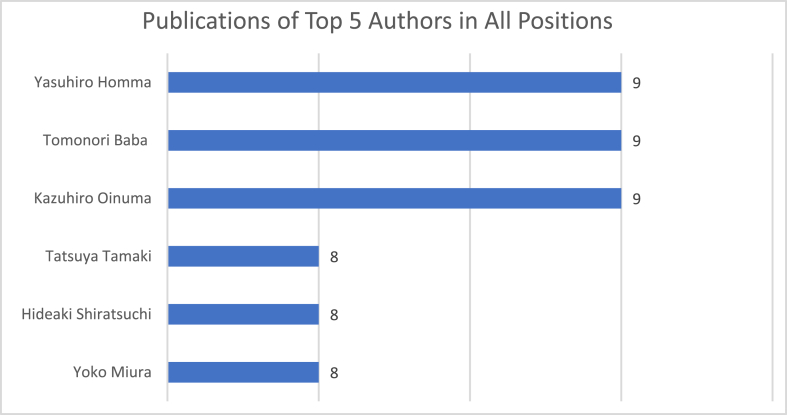
Top 5 authors with most DAA for THR publication in Asian countries.

Fig. 5 shows the top 5 authors with the most publications when they are listed as first authors as well as the number of publication when they are listed as one of the authors. Fig. 6 shows the top 5 authors reporting the DAA for THR in Asian Countries. Top 5 first authors account for 70% from total publication were analyzed in this study.

### 3.4 Most cited

Table 2 listed the titles of the top 10 cited articles and their type of study from PubMed for the DAA for THR in Asian countries.

**Table 2:** Top 10 most cited articles.

Articles	Type of Study	Number of Citations
A Clinical Comparative Study of the Direct Anterior With Mini-Posterior Approach	Cohort	209
Surgical approach and prosthesis fixation in hip arthroplasty world wide	Surgical technique	105
Total Hip Arthroplasty by a Minimally Invasive, Direct Anterior Approach	Surgical technique	92
Comparison of Direct Anterior and Lateral Approaches in Total Hip Arthroplasty	Systematic review/Meta-analysis	55
Lateral femoral cutaneous nerve injury with the direct anterior approach for total hip arthroplasty	Cohort	48
Anatomic Mapping of Short External Rotators Shows the Limit of Their Preservation During Total Hip Arthroplasty	Cadaveric study	42
Comparison of early functional recovery following total hip arthroplasty using a direct anterior or posterolateral approach: a randomized controlled trial	RCT	32
Difference in Stem Alignment Between the Direct Anterior Approach and the Posterolateral Approach in Total Hip Arthroplasty	Surgical technique	32
Surgeons changing the approach for total hip arthroplasty from posterior to direct anterior with fluoroscopy should consider potential excessive cup anteversion and flexion implantation of the stem in their early experience	Cohort	31
Elevation of the Femur in THA Through a Direct Anterior Approach	Cadaveric study	25

### 3.5 Type of study

**Fig. 7:**
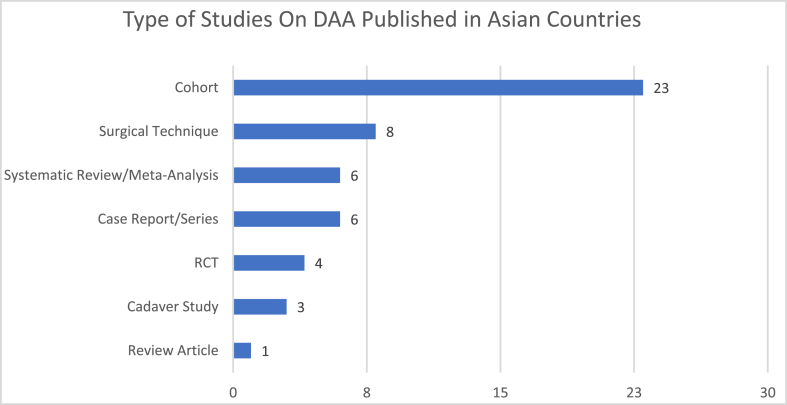
Type of studies on DAA for THR publication in Asian countries.

Fig. 7 shows the types of publication on the DAA for THR by analyzing the article titles. Cohort study was the most commonly published in 23 articles. Surgical technique was found in 8 articles, and cadaver study was in 3 articles. Case report/series was found in 6 articles, and systematic review/meta-analysis was found in 6 articles. Review article was found in 1 publication.

## 4 Discussion

The number of publications on the DAA for THR in Asian countries is still inconsistent. There is even one year without any publications on this topic. Although there were publications in 2020, these were not included in this study, because only publications from completed years were included. However, the average number of publications per year has increased from 2014 onwards compared to the year before 2014.

There was an increasing number of publications from 2011 to 2016, but then fell in 2017 and 2019. This pattern is similar to the publications by Ronfard et al. [13] that show the curve of the Emergence of the therapeutic monoclonal antibody industry (1975—horizon 2020). In their paper, Ronfard et al. [13] has 5 phases of discovery, starting from initial discovery, that are followed by increasing number of research (phase of high expectation), then there will be decreasing number of interests because of the disadvantage/difficulty of the procedure/therapy, this is called phase of dissolution. If this pattern is applied to the papers, the number of publication of DAA for THR in Asian countries will increase again when there is improvement on technique/technology that helps to provide better outcome (phase of product improvements), then later the final phase of technology maturity will be achieved when the technique can be learned and used easily by the surgeons worldwide.

Current data search result from PubMed searches were lack of number of citation details. These data need to be searched indirectly. The results from PubMed sometimes are inconsistent in number and may show different number for the same keyword searched. Another inconsistent results on the data may even be caused by the use of different spelling and terms. For example, “replacement” and “arthroplasty” are two different terms used by authors which have similar meaning although they are very different in spelling.

The most cited article is the article with the title “A Clinical Comparative Study of the Direct Anterior With Mini-Posterior Approach” [14] published by The Journal Of Arthroplasty with the number of citation 209 times. Clinical study especially cohort study was the most commonly published (45%) (Fig. 7). This may show the scrutiny in following up the results of the patient that undergo the DAA for THR. Other studies that published the most is about surgical technique that was found in 8 articles. Case report/series was found in 6 articles, and systematic review/meta-analysis was found in 6 articles. Cadaver study was in 3 articles and review article was found in 1 publication.

Japanese centers published 66,66% of the publication about DAA for THR in Asian countries searched in PubMed. From the top 6 authors with most publications, all of them are from the Japanese centers. From 34 articles published by Japanese centers, 8 articles have impact factor <1. From all publications by Japanese centers, 18, 14, and 1 articles are published by Q1 journal, Q2 journal, and Q4 journal respectively, and 1 journal quality is not listed in www.scimagojr.com.

## 5 Conclusion

There have been relatively small number (12.8%) of articles on the topic of DAA for THR published by Asian countries compared to all research that has been done worldwide. The number of published articles per year is still inconsistent, with one year without any publication on this topic. Searching the data on the DAA for THR in PubMed bestow important and useful information about excellent sources of publication on this topic, including the author/journal that could be followed. The strength of their association with the other researcher may indicate that they are co-workers, having common interest, or teaming up in a collaboratives studies. This is found the most in the articles published by Japanese centers.

## Ethical approval

This is review article, no need ethical approval.

## Funding

The authors declare that this study had no funding resource.

## Author contribution

Sholahuddin Rhatomy and Faiz Alam Rasyid conceived the study. Sholahuddin Rhatomy and krisna yuarno phatama collected data. Sholahuddin Rhatomy, Faiz Alam Rasyid, and krisna yuarno phatama analyzed data. Sholahuddin Rhatomy, Faiz Alam Rasyid, and krisna yuarno phatama prepared and drafted the manuscript. Faiz Alam Rasyid, and krisna yuarno phatama edited manuscript. Sholahuddin Rhatomy and Faiz Alam Rasyid reviewed the manuscript.

## Registration of research studies

This is Review article, no need registration of research studies.

## Guarantor

Sholahuddin Rhatomy,MD.

## Availability of data and material

Data will be provided by request.

## Provenance and peer review

Not commissioned, externally peer reviewed.

## Declaration of competing interest

No potential conflict of interest relevant to this article was reported.

## References

[1] Wang Z., Bao H.W., Hou J.Z. (2019). Direct anterior versus lateral approaches for clinical outcomes after total hip arthroplasty: a meta-analysis. J. Orthop. Surg. Res..

[2] Yoo J Il, Cha Y.H., Kim K.J., Kim H.Y., Choy W.S., Hwang S.C. (2019). Gait analysis after total hip arthroplasty using direct anterior approach versus anterolateral approach: a systematic review and meta-analysis. BMC Muscoskel. Disord..

[3] Jia F., Guo B., Xu F., Hou Y., Tang X., Huang L. (2019). A comparison of clinical, radiographic and surgical outcomes of total hip arthroplasty between direct anterior and posterior approaches: a systematic review and meta-analysis. HIP Int..

[4] Kurtz S., Ong K., Lau E., Mowat F., Halpern M. (2007). Projections of primary and revision hip and knee arthroplasty in the United States from 2005 to 2030. J. Bone Joint Surg..

[5] Wang Z., Hou J zhao, Wu C hua, Zhou Y jiang, Gu X ming, Wang H hong (2018). A systematic review and meta-analysis of direct anterior approach versus posterior approach in total hip arthroplasty. J. Orthop. Surg. Res..

[6] Barrett W.P., Turner S.E., Leopold J.P. (2013). Prospective randomized study of direct anterior vs postero-lateral approach for total hip arthroplasty. J. Arthroplasty.

[7] Talia A.J., Coetzee C., Tirosh O., Tran P. (2018). Comparison of outcome measures and complication rates following three different approaches for primary total hip arthroplasty: a pragmatic randomised controlled trial. Trials.

[8] Takada R., Jinno T., Miyatake K., Hirao M., Kimura A., Koga D. (2018). Direct anterior versus anterolateral approach in one-stage supine total hip arthroplasty. Focused on nerve injury: a prospective, randomized, controlled trial. J. Orthop. Sci..

[9] Free M.D., Owen D.H., Agius P.A., Pascoe E.M., Harvie P. (2018). Direct anterior approach total hip arthroplasty: an adjunct to an enhanced recovery pathway: outcomes and learning curve effects in surgeons transitioning from other surgical approaches. J. Arthroplasty.

[10] Canese K., Weis S. (2013). PubMed: the bibliographic database. NCBI Handb..

[11] Fiorini N., Canese K., Starchenko G., Kireev E., Kim W., Miller V. (2018). Best match: new relevance search for PubMed. PLoS Biol..

[12] Liberati A., Altman D.G., Tetzlaff J., Mulrow C., Gøtzsche P.C., Ioannidis J.P.A. (2009). The PRISMA statement for reporting systematic reviews and meta-analyses of studies that evaluate health care interventions: explanation and elaboration. PLoS Med..

[13] Ronfard V., Vertès A.A., May M.H., Dupraz A., Van Dyke M.E., Bayon Y. (2017). Evaluating the past, present, and future of regenerative medicine: a global view. Tissue Eng. B Rev..

[14] Nakata K., Nishikawa M., Yamamoto K., Hirota S., Yoshikawa H. (2009). A clinical comparative study of the direct anterior with mini-posterior approach. Two consecutive series. J. Arthroplasty.

